# The effect of exposure to MoO_3_-NP and common bean fertilized by MoO_3_-NPs on biochemical, hematological, and histopathological parameters in rats

**DOI:** 10.1038/s41598-022-16022-8

**Published:** 2022-07-15

**Authors:** Eman E. Shaban, Dina M. Salama, Mahmoud E. Abd El-Aziz, Khadiga S. Ibrahim, Soad M. Nasr, Hassan M. Desouky, Hagar F. H. Elbakry

**Affiliations:** 1 Environmental & Occupational Medicine Department, National Research Centre, P.O. 12622, Giza, Egypt; 2 Vegetable Research Department, National Research Centre, P.O. 12622, Giza, Egypt; 3 Polymers and Pigments Department, National Research Centre, P.O. 12622, Giza, Egypt; 4 Parasitology and Animal Diseases Department, National Research Centre, P.O. 12622, Giza, Egypt; 5 Animal Reproduction and Artificial Insemination Department, National Research Centre, P.O. 12622, Giza, Egypt; 6 Nutrition and Food Sciences Department, National Research Centre, P.O. 12622, Giza, Egypt

**Keywords:** Biochemistry, Environmental sciences

## Abstract

Nanotechnologies has been used to introduce several beneficial tools in the agricultural field. Herein, the effect of molybdenum oxide nanoparticles (MoO_3_-NPs) was investigated by evaluating the hematological, biochemical, and histopathological parameters in rats orally exposed to MoO_3_-NPs or fed common beans (CB) fertilized by MoO_3_-NPs. In the first study, 18 rats were randomly divided into 3 groups: G1 (control group) was given water orally, while G2 and G3 were administered 10 and 40 ppm MoO_3_-NPs by oral gavage tube, respectively. There was a significant increase in the levels of alanine aminotransferase (ALT), albumin, and total protein; however, there was a a significant decrease in body weight change (BWC), alkaline phosphatase (ALP), lactate dehydrogenase (LDH), creatinine, creatine kinase–MB (CK-MB), thyroid-stimulating hormone (TSH), free triiodothyronine (FT3), and testosterone levels in G3 compared to G1. In the second study, 24 rats were divided into 4 groups: the control (C) group was fed a balanced diet, and three groups were fed on a balanced diet plus 10% CB that was fertilized with 0, 10, and 40 ppm MoO_3_-NPs, resulting in nCB, CB10, and CB40 groups, respectively. This revealed a significant increase in BWC and total food intake (TFI) but a significant decrease in relative kidney weight in all the CB groups compared to the control group. In CB10 and CB40 groups ALT, LDH, TSH, FT3, and testosterone levels were significantly lower than the respective levels in the control group. We concluded that high doses of MoO_3_-NPs caused more side effects than low doses in both experiments.

## Introduction

Molybdenum (Mo) is an essential transition metal for plants, animals, and human health. Animals and plants can be harmed either by excess or lack of Mo^1^. Once Mo enters the human body, it is primarily stored in the liver as molybdenite. The molybdopterin cofactor consists of a Mo ion and is essential for the activity of enzymes such as sulfite oxidase, xanthine oxidase, and aldehyde oxidase^2^. Some researchers have focused on the biological effects of MO on nonalcoholic fatty liver disease (NAFLD)^3^.

According to Ale- Ebrahim et al.^4^ sodium molybdate reduced triglyceride (TG), and cholesterol levels and minimized oxidative damage in the hepatic cells Furthermore, it reduced hepatic steatosis and demonstrated anti-fibrotic properties in rats. Moreover, the metabolism of phosphorus, copper, sulfur, zinc, iodine, and potassium was affected by Mo^5^. It was demonstrated that Mo complexes were effective against diabetes^6^. Moreover, it was demonstrated that sodium molybdate had an insulin-mimicry activity and improved immune dysfunction in diabetic rats^7^. Burguera and Burguera^8^ discovered that a high intake of MO-rich grains, legumes, and seeds could lead to the deposition of Mo in the soft tissues and joints, resulting in arthritic symptoms. Mo poisoning has also been linked to severe gastrointestinal irritation with diarrhea and ruminant’s coma and death from cardiac failure.

Nanotechnological innovations have led to the development of a wide variety of nanoparticles (NPs) with large surface area and high bioactivity in the cells^9,10^. The toxicity of NPs is affected by particle size, chemical nature, surface chemistry, and morphology. As a result, it is critical to have access to all necessary information and clarifications to assess the risks associated with the NPs^11,12^. According to the literature, exposure to ultrafine particles (< 100 nm) could induce tissue inflammation and pulmonary damage, lung tumors, and fibrosis^13^. However, the generation of reactive oxygen species (ROS) is mainly associated with the toxicity of NPs. ROS accumulation triggers various physiological and cellular events, including inflammation, cellular stress, DNA damage, and apoptosis^14^. Mo-NPs have been previously used in many applications such as electronics, glass manufacturing, electrochemical capacitors, coatings, plastics, and X-ray tubes^15^. Moreover, Mo-NPs have agricultural applications, where they improve microbiological activity in the rhizosphere of chickpeas and increase their length, diameter, and root circumference^16^. However, some studies have reported the presence of Mo-NPs in industrial and agricultural wastes and their effect on animal and human health^17^. Mo-NPs were found to increase the content of intracellular antioxidant glutathione to protect cells against oxidants, thus playing a role against oxidative stress^18^.

The Common bean (CB) is a member of the leguminous family. It is considered one of the most vital vegetable crops sowed in Egypt for exportation. It is enriched with a high protein source, accounting for 22%–27% of seed weight^19,20^. Each 100 g of CB supplied about 20 g of protein in humans, nearly 20% of the endorsed quotidian assimilation for an average adult^21^. In the recent decade, CB has been identified as a valuable food ingredient for over half a billion people on account of its high content of bioactive compounds, such as polyphenols, resistant starch, oligosaccharides, and bioactive peptides nutrients^22^. It also contains complex carbohydrates, unsaturated fats, vitamins, minerals, and several antioxidants^23^. In addition, the processing of legumes is essential for eliminating or reducing anti-nutritive factors, for example, polyphenols and flavonoids^24^. The anti-nutrient factors are inhibitors for growth like tannins that inhibit the digestibility of proteins and phytic acid, decreasing mineral bioavailability^25^.

This study aimed to investigate the effect of MoO_3_-NPs on the consumers who eat the CB fertilized by MoO_3_-NP and farmers exposed to MoO_3_-NPs as nanofertilizer. The study focused on evaluating nutritional parameters and liver, kidney, and heart functions. In addition, we studied the impact of MoO_3_-NPs on the thyroid and testosterone hormones. Hematology assays were also performed. Furthermore, histological examinations of the liver, kidney, heart, and testicular tissues were performed.

## Material and methods

### Materials

Common bean (CB) seeds were delivered from Agricultural Research Centre, Egypt. Citric acid and ammonium molybdate were obtained from Sigma-Aldrich Company.

### Preparation of molybdenum oxide nanoparticles (MoO_3_-NPs) via sol–gel method

A solution (100 ml) of ammonium molybdate (11.6 g) and citric acid (3.8 g) was prepared. Thereafter that ammonium hydroxide was added under continuous stirring untill pH was 7. The previous solution was heated in a furnace for an hour at 250 °C, and then the obtained powder was burned for 120 min at 500 °C^26^.

### Characterization

A transmission electron microscope (TEM; model JEM-1230, Japan) was utilized to study the morphological and particle size of MoO_3_-NPs. Furthermore, the XRD pattern of MoO_3_-NPs was achieved from a Diano X-ray diffractometer using CoKα radiation source energized at 45 kV and a Philips X-ray diffractometer (PW 1930 generator, PW 1820 goniometer) with CuK radiation source (λ = 0.15418 nm).

### Agricultural experiment layout

The seeds of CB were sown in clay soil at 120 kg/ha in the first week of March in El-Monifia governorate, Egypt, during two seasons, 2018 and 2019. After 20 days of planting seeds, the plants were sprayed with MoO_3_-NPs at a concentration of 0, 10, 20, 30, and 40 ppm, as previously reported^27^.

### Nutritional experiment

Common bean seeds were split into three types depending on the dose of MoO_3_-NPs (0, 10, and 40 ppm) that were utilized as a foliar application during the cultivation of the CB plants, to nCB, CB10, and CB40, respectively. The seeds were firstly cleaned from dust and foreign matter manually and then washed with water. The seeds of each sample were soaked for four hours in distilled water (1 kg/5 l) at 25 °C after that they were washed and then cooked in distilled water at 100 °C (standard cooking)^28^. After that, they were dried at 60 °C before being ground through a 40 mesh screen. This flour was utilized in the preparation of experimental diets.

### Experimental procedures

**Scheme 1:**
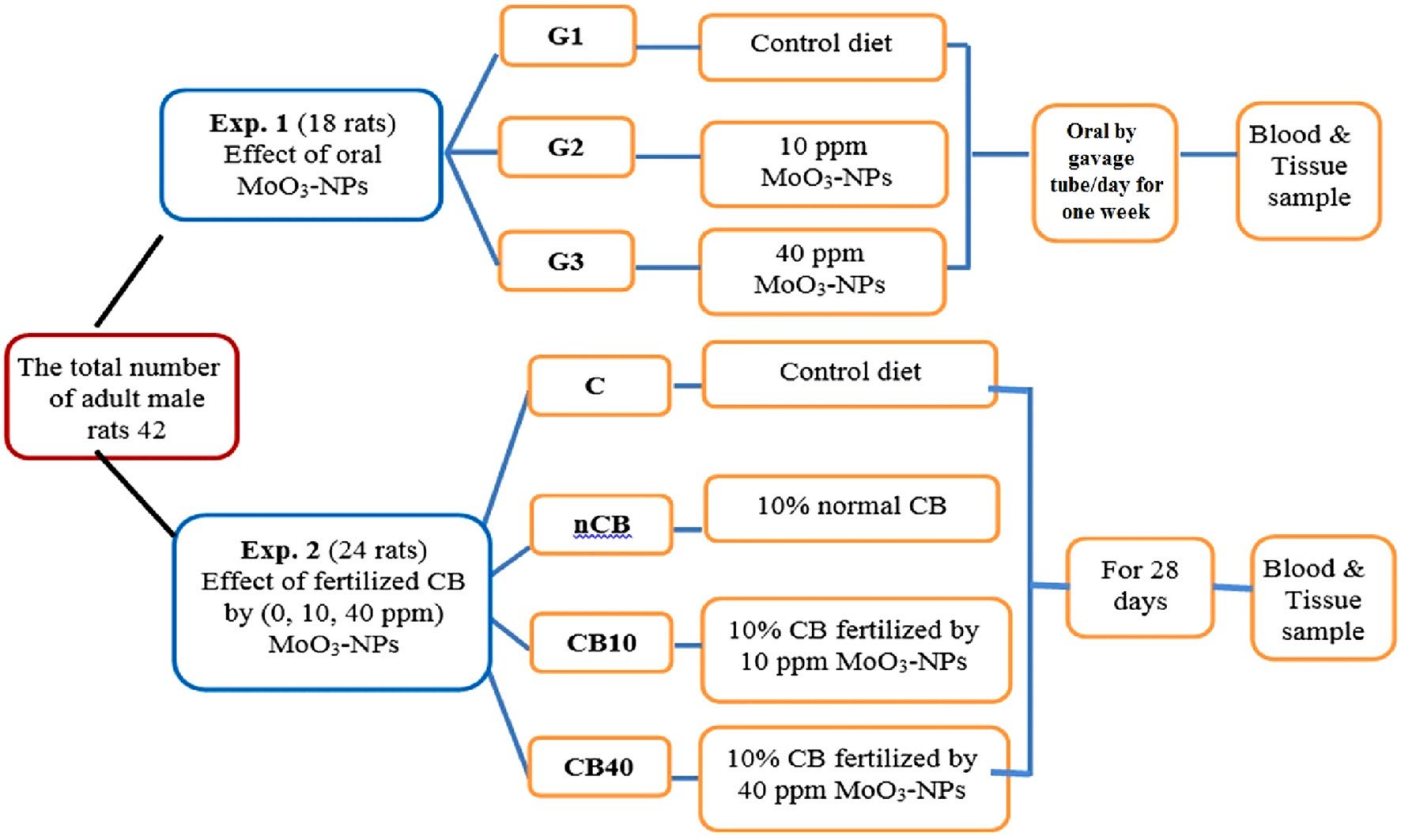
Experimental design chart.

Forty-two male albino rats weighing 80–130 g were purchased from the animal house of the National Research Centre (NRC), Giza, Egypt. Animals were kept individually in stainless steel cages at a temperature (25 °C  ± 2 °C) under a 12 h dark–light cycle with proper water and food supply for 1 week before the experiments for acclimatization. All methods, experimental procedures, and animal comfort were performed by and under the Animal Care and Ethics Committee (Registration Number 16457) of the National Research Centre and the ARRIVE guidelines. We conducted two separate experiments (Scheme 1).

The first experiment, which lasted 1 week, showed the influence of oral exposure to MoO_3_-NPs on rats. Eighteen rats were split randomly into three groups of six rats each, and all the rats were nourished on a diet prepared in agreement with the AOAC guidelines^29^. At the same time, the salt and vitamin mixtures were made according to Williams and Briggs^30^ and Morcos^31^, respectively. The first group (G1) is a control group in which each rat was given deionized water orally. The second (G2) and third (G3) groups were given 1 ml of 10 and 40 ppm of suspended MoO_3_-NPs, respectively, orally by gavage tube/day for 1 week. It should be noted that the reason for using 10 and 40 ppm of suspended MoO_3_-NPs was the minimum and maximum value, respectively, used in the fertilization of CB.

The second experiment, which lasted 28 days, investigated the effect of fertilized CB on rats. Twenty-four rats were randomly divided into four groups each with six rats: The first group was the control, nourished with an equiponderant diet (C). The second group was a positive control that received an equiponderant diet having 10% of normal CB at the expense of carbohydrates (nCB). The third group received an equiponderant diet containing 10% treated CB sprayed with 10 ppm MoO_3_-NPs (CB10). The last group was nourished on an equiponderant diet containing 10% of treated CB sprayed with 40 ppm MoO_3_-NPs (CB40).

The animal body weight, body weight change, total food intake (TFI), and food efficiency ratio (FIR) were monitored. After the experimental period, all groups were fasted overnight (12 h). Blood specimens were attained from the retro-orbital vein under light anesthesia on ethylene diamine tetraacetic acid and into heparinized tubes. The first part was utilized for the hematological testing, while the other was centrifuged at 4000 rpm for 10 min to separate the plasma. Plasma was kept at -20ºC for various biochemical analyses.

### Hematological examination

The complete blood profile for all the groups was determined using a hematological analyzer (MEDONIC, S.E 12613, Sweden). Red blood cells (RBCs) count, hematocrit (HCT), hemoglobin (Hb) concentration, red cell indices, mean corpuscular hemoglobin concentration (MCHC), mean corpuscular hemoglobin (MCH), mean corpuscular volume (MCV), platelets (PLT), and white blood cells (WBCs) and its differential lymphocytes, granulocytes, and monocytes were all measured.

### Biochemical analyses

The following parameters were analyzed in plasma using a spectrophotometric method with commercial kits provided by Biodiagnostic, Egypt. Liver function was assessed by measuring aspartate aminotransferase (AST) and alanine aminotransferase (ALT) using a colorimetric method based on the principles of Reitman and Frankl^32^. The activity of plasma alkaline phosphatase (ALP) was measured using the Belfield and Goldberg^33^ method and the activity of lactate dehydrogenase (LDH) was measured using the Zimmerman and Henery^34^ method. According to Doumas et al.^35^, the synthetic function of the liver was determined by measuring plasma albumin concentration. Total protein was calculated using the Rheinhold (1953) method. Meanwhile, renal function was evaluated by determining plasma urea^36^, uric acid (UA)^37^, and creatinine levels^38^. Blood glucose was instantaneously determined using a glucometer. Thyroid-stimulating hormone (TSH)^39^, free triiodothyronine (FT3)^40^, and testosterone hormone were measured using an ELISA kit purchased from Life Diagnostics (West Chester, Pa) and used following the manufacturer’s instructions. Also, creatine kinase-MB (CK-MB) was measured using the Urdal and Landaas^41^ method.

### Histopathological study

The liver, kidneys, heart, testes, and thyroid gland were dissected and immediately fixed in 10% formalin saline for 24 h. The specimens were washed with tap water, dehydrated in ascending grades of ethanol, cleared in xylene, and fixed in paraffin wax (melting point 55–60 °C). For histopathological examination, 6 mm thick sections were prepared and stained with haematoxylin and eosin stain^42^.

### Statistical analysis

SPSS (version 20) statistical package was used for the analysis of data. Data were designated as a mean ± standard error of the mean (SE). To compare the statistical differences between groups, a one-way ANOVA test was utilized followed by the least significant difference (LSD) for all variables. The statistical significance criterion was set at *p* ≤ 0.05.

### Ethical approval

We confirm that all experimental protocols were approved by the Animal Care and Ethics Committee (Registration Number 16457) of the National Research Centre, Egypt. We confirm that all methods were carried out in accordance with relevant guidelines and regulations. All methods, experimental procedures, and animal comfort were performed per the ARRIVE guidelines.

## Results

**Figure 1:**
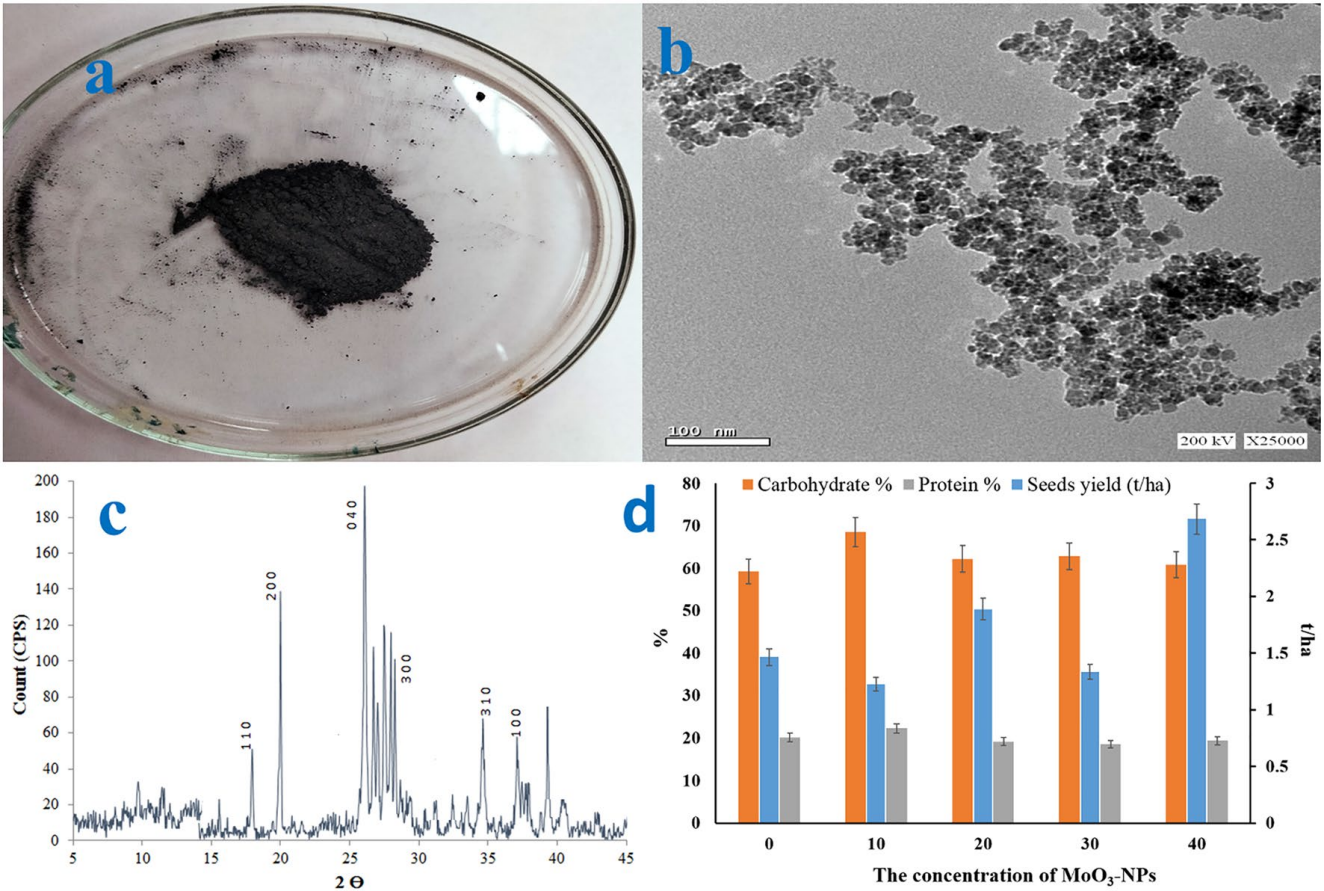
Photographic (**a**), TEM image (**b**) and XRD pattern (**c**) of MoO_3_–NPs, as well as (**d**) the seeds yield (t/ha), and the content of carbohydrate and protein (%) in the CB, the average of the two seasons 2018 and 2019.

Figure 1a showed the degetal image of MoO_3_-NPs powder. Figure 1b showed the TEM image of MoO_3_-NPs, with a particle size of less than 50 nm. Figure 1c displayed the XRD pattern of MoO_3_-NPs with peaks at 2θ = 17.9, 19.9, 26.1, 34.5, and 39.3 corresponding to planes (1 1 0), (2 00), (0 4 0), (3 1 0), and (1 0 0), respectively, which emphasizes on the pureness of MoO_3_-NPs^43^. Figure 1d demonstrated that the seed yield increased by 83.2% with 40 ppm while the best content of carbohydrate and protein content in CB seeds was shown at 10 ppm MoO_3_–NPs.

### Nutritional and biochemical parameters for the first experiment

Table 1 demonstrated that G2 and G3 had a substantial variation in the BWC compared to G1. When the initial body weight (IBW) was compared to the final body weight (FBW) of rats, the G2 and G3 showed a reduction of 3.5% and 4.5%, respectively.

**Table 1:** Nutritional changes and relative organ weight for various studied groups (1 week).

Groups	G1	G2	G3	ANOVA	
				F-ratio	*P*-value
IBW (g)	103.8 ± 5.5	103.8 ± 4.9	104 ± 5.8	0.000	NS
FBW (g)	108.7 ± 5.7	100.2 ± 4.8	99.3 ± 6.2	0.857	–
BWC (g)	4.8 ± 0.5^bc^	3.7 ± 1.6^a^	4.7 ± 3.3^a^	5.960	0.012
Relative liver weight (g%)	4.068 ± 0.35	4.292 ± 0.29	3.814 ± 0.17	0.725	NS
Relative kidney weight (g%)	1.009 ± 0.03	1.105 ± 0.09	1.112 ± 0.06	0.991	NS
Relative spleen weight (g%)	0.501 ± 0.04	0.548 ± 0.03	0.528 ± 0.07	0.217	NS
Relative testis weight (g%)	1.494 ± 0.15	1.584 ± 0.2	1.542 ± 0.17	0.068	NS
Relative heart weight (g%)	0.505 ± 0.03	0.535 ± 0.02	0.534 ± 0.03	0.412	NS

Values are expressed as mean ± S.E; value (a) is significantly different as compared to G1, value (b) is significantly different as compared with the G2, value (c) is significantly different as compared with the G3.

*IBW* initial body weight, *FBW* final body weight, *BWC* body weight change.

All organs of the rats in G2 had a nonsignificant increase in the relative weight compared with the relative weight of control rats (Table 1). Moreover, G3 had a nonsignificant reduction in liver relative to its weight and a nonsignificant increase in the relative weight of the kidney, spleen, testes, and heart compared with the relative weight of control rats.

Table 2 shows the function of the liver and kidneys plus the blood glucose levels. G3 had a considerable increase in ALT, albumin, and total protein compared to the respective levels in the control group, while G2 revealed a considerable increase in plasma urea levels. Meanwhile, G3 showed a substantial decrease in ALP, LDH, creatinine, CK-MB, TSH, Ft3, and testosterone compared to the respective levels in the control group. Furthermore, when compared to the control group, most of these biochemical analyses revealed a nonsignificant alteration in G2.

**Table 2:** Biochemical parameters for 1 week of oral exposure to MoO_3_-NPs.

Groups	G1	G2	G3	ANOVA	
				F-ratio	*P*-value
AST (U/ml)	35 ± 2.5	37.2 ± 3.2	37.5 ± 2.9	0.224	NS
ALT (U/ml)	9 ± 0.7^c^	9.9 ± 1.1	12.1 ± 0.9^a^	2.915	NS
ALP (IU/l)	246 ± 14.5^c^	234 ± 11.2	209 ± 8.6^a^	2.631	NS
LDH (U/l)	592 ± 16.4^bc^	578 ± 11.4^c^	434 ± 18.2^ab^	31.6	≤ 0.001
Albumin (g/dl)	2.74 ± 0.10^bc^	2.69 ± 0.19^c^	3.32 ± 0.14^ab^	5.437	≤ 0.05
Total protein (g/dl)	5.62 ± 0.25^bc^	5.62 ± 0.52^c^	7.17 ± 0.31^ab^	5.448	≤ 0.05
Albumin–globulin ratio	0.98 ± 0.086	0.96 ± 0.091	0.898 ± 0.099	0.214	NS
Urea (mg/dl)	32.2 ± 1.5	42.3 ± 1.3^ac^	34.3 ± 1.5	13.5	≤ 0.001
UA (mg/dl)	2.98 ± 0.16	3.13 ± 0.24	3.51 ± 0.27	1.41	NS
Creatinine(mg/dl)	0.702 ± 0.03^bc^	0.605 ± 0.04^c^	0.464 ± 0.04^ab^	10.2	0.002
BUN	15.03 ± 1.5^b^	19.8 ± 1.3^ac^	16.04 ± 1.5^b^	13.5	≤ 0.001
Glucose (mg/dl)	67.8 ± 8.9	68.5 ± 6.6	61.3 ± 3.5	0.345	NS
CK-MB (U/l)	96.2 ± 8.02^c^	80.2 ± 4.6	77.5 ± 5.4^a^	2.664	NS
TSH (µg/dl)	1.08 ± 0.02^c^	0.923 ± 0.08	0.862 ± 0.05^a^	4.156	0.037
FT3 (pmol/l)	8.8 ± 0.76^c^	7.9 ± 0.63	6.9 ± 0.36^a^	2.496	NS
Testosterone (ng/l)	1.37 ± 0.1^c^	1.192 ± 0.04	1.03 ± 0.06^a^	5.542	0.016

Values are expressed as mean ± S.E; value (a) is significantly different as compared with the G1, value (b) is significantly different as compared with the G2, value (c) is significantly different as compared with the G3.

*ALT* alanine aminotransferase, *AST* aspartate aminotransferase, *ALP* alkaline phosphatase, *LDH* lactate dehydrogenase, *CK-MB* creatine kinase–MB, *TSH* thyroid-stimulating hormone, *FT3* freetriiodothyronine, *UA* uric acid, *BUN* blood urea nitrogen.

Table 3 demonstrates the difference in hematological parameters in rats. The WBCs and the differential blood count (lymphocyte, monocyte, and granulocyte) were significantly lower in G2 than in G1. In addition, lymphocytes and monocytes were substantially more in G3 than in G2. In addition, we found that RBC and hemoglobin levels were substantially higher in G3 than in G1 and G2. The platelet and RBC indices did not significantly alter.

**Table 3:** Hematological parameters for various experimental groups (1 week).

Groups	G1	G2	G3	ANOVA	
				F-ratio	*P*-value
RBCs (10^6^/µl)	6.998 ± 0.27^c^	6.83 ± 0.39^c^	8.13 ± 0.29^ab^	4.91	0.023
WBCs (10^3^/µl)	13.8 ± 0.8^bc^	9.7 ± 0..6^a^	11.3 ± 0.8^a^	8.22	0.004
Hemoglobine (g/dl)	12.2 ± 0.5^c^	12.4 ± 0.6^c^	14.1 ± 0.5^ab^	3.367	NS
Hematocrate (%)	38.7 ± 1.2^c^	38.98 ± 1.8	43.57 ± 1.6^a^	3.099	NS
Platelet (10^3^/µl)	725 ± 34	673 ± 26	692 ± 52	0.437	–
MCV(fl)	55.4 ± 1.2	55.96 ± 1.2	53.6 ± 0.93	1.23	–
MCH (pg)	17.4 ± 0.24	17.8 ± 0.22	17.2 ± 0.18	1.767	–
MCHC (g/dl)	31.6 ± 0.51	31.9 ± 0.34	32.3 ± 0.27	0.793	–
Lymphocyte	8.2 ± 0.6^bc^	5.2 ± 0.4^a^	8.1 ± 0.6^b^	10.74	0.001
Monocyte	1.1 ± 0.03^bc^	0.8 ± 0.07^a^	0.98 ± 0.09^b^	5.23	0.019
Granulocyte	4.5 ± 0.43^bc^	3.4 ± 0.24^ac^	2.23 ± 0.24^ab^	11.91	0.001

Values are expressed as mean ± S.E, value (a) is significantly different as compared with G1, value (b) is significantly different as compared with G2 and value (c) is significantly different as compared with the G3.

*RBCs* Red blood cells count, *MCHC* mean corpuscular hemoglobin concentration, *MCH* mean corpuscular hemoglobin, *MCV* mean corpuscular volume, *WBCs* white blood cells.

**Figure 2:**
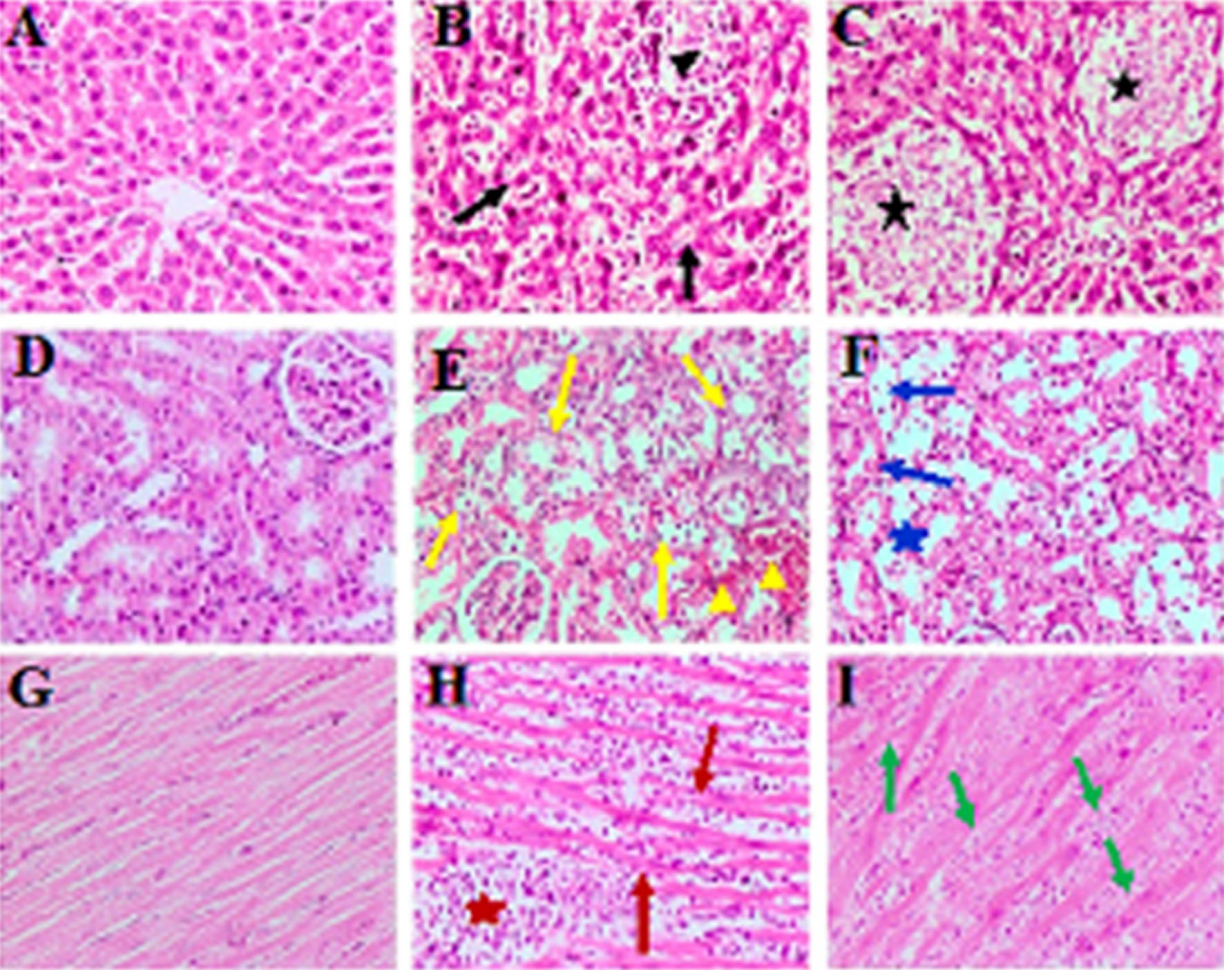
Photomicrographs of the liver (**A**–**C**), kidney (**D**–**F**), and heart (**G**–**I**) from rats of groups G1, G2, and G3, respectively, (H&E, × 200). Degeneration and dissociation of hepatic cords (black arrows), dilation of hepatic sinusoids and inflammatory cells (black head arrow), multiple areas of hepatic cell necrosis (black asterisk), necrotic changes of tubular epithelium (yellow arrows), focal area of hemorrhage (yellow head arrow), necrosis of tubular epithelium (blue arrows), exfoliation of necrotic cells with dilatation of renal tubules (blue asterisk), diffuse infiltration of mononuclear cells (brown asterisk), hyaline degeneration (brown arrow), Zenkers necrosis (green arrow).

The liver, kidneys, and heart of control rats (G1) had no microscopic alterations (Fig. 2A,D, G). However, in G2, the liver showed degeneration and dissociation of the hepatic cords associated with marked dilation of hepatic sinusoids and inflammatory cells and a large aggregation of mononuclear inflammatory cells, mainly lymphocytes in the portal area (Fig. 2B). The kidneys revealed necrotic modulations in the epithelial lining of renal tubules with focus desquamation of necrotic cells into the lumen. In addition, several bleeding regions and the renal blood vessels seemed dilated and congested (Fig. 2E). Moreover, mononuclear inflammatory cell infiltration was mainly observed involving the lymphocytes among the necrotic renal tubules. Meanwhile, in the heart, focal and spread leakage of mononuclear cells, primarily lymphocytes and macrophages associated with hyaline degeneration of myofibers, were observed (Fig. 2H).

However, in G3, the liver showed multiple areas of hepatic cell necrosis associated with lymphocytic cell infiltration, necrosis, and extensive dissociation of hepatic cords (Fig. 2C). The kidneys demonstrated severe necrotic changes in the epithelium lining of renal tubules, shedding necrotic cells into the lumen with dilatation of tubules in many areas of the renal cortex. Moreover, severe hemorrhage and congestion of renal blood vessels were observed (Fig. 2F). The heart showed multiple areas of coagulative necrosis (Zenker’s necrosis) of the myocardial fibers, which were swollen with a loss of cross-striation pattern and had an eosinophilic hyaline appearance. Moreover, the focal aggregation permeated mononuclear inflammatory cells in the myocardium and pericardium was observed (Fig. 2I).

### Nutritional and biochemical parameters for the second experiment

Table 4 demonstrates the variations in various nutritional parameters. Compared to the control group, nCB, CB10, and CB40 exhibited substantial growth in FBW, BWC, and TFI. TFI was significantly reduced in both CB10 and CB40 groups than in the nCB group. In addition, there was a substantial increase in FIR in the CB10 group than in the control.Table 4 also shows a nonsignificant decrease in the relative weight of the liver and heart. The relative weight of testes was nonsignificantly increased in all CB groups than in the control group. The relative weight of the kidney was considerably lower in the nCB, CB10, and CB40 groups than in the control group. Moreover, the relative weight of the spleen was nonsignificantly reduced in the nCB and CB40 groups than in the control group.

**Table 4:** Nutritional changes and relative organ weight for various studied groups (1 month).

Groups	C	nCB	CB10	CB40	ANOVA	
					F-ratio	*P*-value
IBW (g)	102.2 ± 4.3	101.8 ± 5.2	101 ± 3.8	101.7 ± 6.4	0.009	NS
FBW (g)	133 ± 4^bcd^	182 ± 5^a^	176 ± 10^a^	164 ± 9^a^	8.898	= 0.001
BWC (g)	31 ± 2.4^bcd^	80 ± 9.7^a^	75 ± 12.5^a^	62 ± 12.3^a^	4.788	≤ 0.05
TFI (g)	208 ± 34^bcd^	467 ± 21.7^a^	332 ± 42^ab^	370 ± 25^ab^	17.147	≤ 0.001
FER	0.14 ± 0.02	0.17 ± 0.02	0.22 ± 0.02^a^	0.17 ± 0.03	2.252	NS
Relative liver wt. (g%)	4.001 ± 0.50	3.295 ± 0.14	3.274 ± 0.12	3.337 ± 0.19	1.553	NS
Relative kidney wt. (g%)	1.122 ± 0.06^bcd^	0.925 ± 0.04^a^	0.858 ± 0.03^a^	0.963 ± 0.02^a^	7.026	≤ 0.01 +
Relative heart wt. (g%)	0.461 ± 0.04	0.416 ± 0.02	0.415 ± 0.03	0.409 ± 0.01	0.809	NS
Relative spleen wt. (g%)	0.507 ± 0.05	0.434 ± 0.04	0.347 ± 0.03^a^	0.421 ± 0.03	2.634	NS
Relative testes wt. (g%)	1.074 ± 0.25	1.223 ± 0.06	1.243 ± 0.05	1.292 ± 0.11	0.501	NS

Values are expressed as mean ± S.E, value (a) is significantly different as compared with the control group, value (b) is significantly different as compared with nCB, value (c) is significantly different as compared with CB10, and value (d) is significantly different as compared with CB40.

*IBW* initial body weight, *FBW* final body weight, *BWC* body weight change, *TFI* total food intake, *FER* food efficiency ratio, *wt* weight.

Table 5 shows the functioning of the liver and kidneys, blood glucose, and hormone levels. Compared to the control group, the AST levels were significantly higher in the nCB groups and nonsignificantly increased in the two treated groups (CB10 and CB40). All CB groups had significantly higher ALP and lower ALT levels than the respective levels in the control group. Lactate dehydrogenase and CK-MB levels were significantly lower in the CB10 and CB40 groups. At the same time, total protein was considerably lower in the nCB and CB10 groups than in the control group. Moreover, the nCB group had a significant increase in urea and creatinine compared to the control group, and this increase was reduced in both the CB10 and CB40 groups than in the nCB group.

**Table 5:** Biochemical parameters for various experimental groups (1 month).

Groups	C	nCB	CB10	CB40	ANOVA	
					F-ratio	*P*-value
AST (U/ml)	37.2 ± 3.32^b^	47.5 ± 2.78^a^	39 ± 1.75	42.5 ± 4.25	2.075	NS
ALT (U/ml)	13.0 ± 0.7^bcd^	9.0 ± 1.03^a^	8.3 ± 1.20^a^	8.1 ± 1.15^a^	4.830	0.011
ALP (IU/l)	120 ± 3.03^bcd^	154 ± 9.83^a^	149 ± 6.15^a^	146 ± 8.9^a^	4.134	0.020
LDH (U/l)	691 ± 10.1^cd^	658 ± 20.9^d^	615 ± 24.5^a^	590 ± 29.2^ab^	4.062	0.021
Albumin (g/dl)	3.26 ± 0.09	2.90 ± 0.14^d^	2.98 ± 0.18^d^	3.67 ± 0.14^bc^	5.992	0.004
Total protein (g/dl)	6.73 ± 0.28^bc^	5.93 ± 0.12^a^	5.22 ± 0.32^ad^	6.04 ± 0.25^c^	5.980	0.004
CK-MB (U/l)	88.8 ± 6.2^cd^	84.0 ± 5.7^d^	70.7 ± 6.4^a^	64.2 ± 5.2^ab^	3.734	≤ 0.05
Urea (mg/dl)	33.7 ± 2.3^b^	43.9 ± 1.8^acd^	33.4 ± 1.4^b^	30.1 ± 2.2^b^	9.517	≤ 0.001
UA (mg/dl)	2.35 ± 0.10	2.81 ± 0.19	2.41 ± 0.33	2.36 ± 0.24	0.920	NS
Creatinine (mg/dl)	0.526 ± 0.0^bc^	0.689 ± 0.03^ad^	0.673 ± 0.03^ad^	0.521 ± 0.03^bc^	12.254	≤ 0.001
BUN	15.7 ± 1.07^b^	20.5 ± 0.82^acd^	15.6 ± 0.66^b^	14.1 ± 1.01^b^	9.517	≤ 0.001
Glucose (mg/dl)	51.7 ± 2.6^bcd^	78.7 ± 3.9^ad^	70.2 ± 2.8^a^	63.0 ± 2.6^ab^	14.543	≤ 0.001
TSH (µg/dl)	1.024 ± 0.04^cd^	0.993 ± 0.06^cd^	0.734 ± 0.04^abd^	0.578 ± 0.02^abc^	24.168	≤ 0.001
FT3 (pmol/l)	8.83 ± 0.60^bcd^	7.92 ± 0.44^acd^	7.35 ± 0.53^ab^	6.18 ± 0.40^ab^	4.993	= 0.01
Testosterone (ng/ml)	2.03 ± 0.2^cd^	1.99 ± 0.1^cd^	1.52 ± 0.09^ab^	1.48 ± 0.09^ab^	4.065	≤ 0.05

Values are expressed as mean ± S.E, value (a) is significantly different as compared with the control group, value (b) is significantly different as compared with nCB, value (c) is significantly different as compared with CB10, and value (d) is significantly different as compared with CB40.

*ALT* alanine aminotransferase, *AST* aspartate aminotransferase, *ALP* alkaline phosphatase, *LDH* lactate dehydrogenase, *CK-MB* creatine kinase–MB, *TSH* thyroid-stimulating hormone, *F T3* free triiodothyronine, *UA* uric acid, *BUN* blood urea nitrogen.

Furthermore, compared to the control group, the blood glucose concentrations in the CB groups were considerably higher. However, compared to nCB, blood glucose concentrations were minimum in the CB10 group and significantly less in the CB40 group. Moreover, when compared to control, TSH, FT3, and testosterone levels were significantly lower in the CB10 and CB40 groups, and the latter hormones were significantly lower in the CB40 group than in the CB10 group.

Table 6 demonstrates the alterations in the hematological parameters between the CB groups and the control. RBC, hemoglobin, and hematocrit were considerably higher in the CB10 and CB40 groups than in the control group. Platelets were also significantly less in the CB groups than in the control group. Furthermore, the granulocyte and monocyte counts were significantly lower in the CB10 and CB40 groups than in the control group, and the monocyte count in the CB40 group was much lower. Other hematological parameters showed insignificant changes.

**Table 6:** Hematological parameters for various experimental groups (1 month).

Groups	C	nCB	CB10	CB40	ANOVA	
					F-ratio	*P*-value
RBCs (10^6^/µl)	7.9 ± 0.15^cd^	8.1 ± 0.20^c^	8.9 ± 0.18^ab^	8.5 ± 0.24^a^	5.052	≤ 0.01
WBCs(10^3^/µl)	17.2 ± 0.9	15.4 ± 1.3	14.6 ± 0.7	15.1 ± 0.7	1.522	NS
Hemoglobin (g/dl)	14.2 ± 0.11^cd^	14.4 ± 0.14^cd^	15.6 ± 0.20^ab^	15.2 ± 0.27^ab^	12.535	≤ 0.001
Hematocrate (%)	40.1 ± 0.75^cd^	40.8 ± 0.37^cd^	44.3 ± 0.55^ab^	42.9 ± 0.63^ab^	10.68	≤ 0.001
Platelet (10^3^/µl)	791 ± 27^bcd^	705 ± 35^a^	697 ± 28^a^	625 ± 26^a^	5.37	≤ 0.01
MCV(fl)	50.4 ± 1.1	50.4 ± 1.2	49.8 ± 0.8	50.4 ± 1.2	0.056	NS
MCH (pg)	17.9 ± 0.35	17.7 ± 0.38	17.6 ± 0.16	17.8 ± 0.33	0.143	–
MCHC (g/dl)	35.5 ± 0.3	35.2 ± 0.23	35.3 ± 0.17	35.5 ± 0.25	0.267	–
Lymphocyte	13.1 ± 0.8	12.2 ± 1.1	11.4 ± 0.7	11.95 ± 0.7	0.704	–
Monocyte	0.50 ± 0.04^d^	0.40 ± 0.03	0.45 ± 0.06	0.35 ± 0.03^a^	2.397	–
Granulocyte	3.55 ± 0.36^cd^	2.86 ± 0.20	2.56 ± 0.17^a^	2.83 ± 0.17^a^	3.028	≤ 0.05

Values are expressed as mean ± S.E, value (a) is significantly different as compared with the control group, value (b) is significantly different as compared with nCB, value (c) is significantly different as compared with CB10, and value (d) is significantly different as compared with CB40.

*RBCs* Red blood cells, *MCHC* mean corpuscular hemoglobin concentration, *MCH* mean corpuscular hemoglobin, *MCV* mean corpuscular volume, *WBCs* white blood cells.

**Figure 3:**
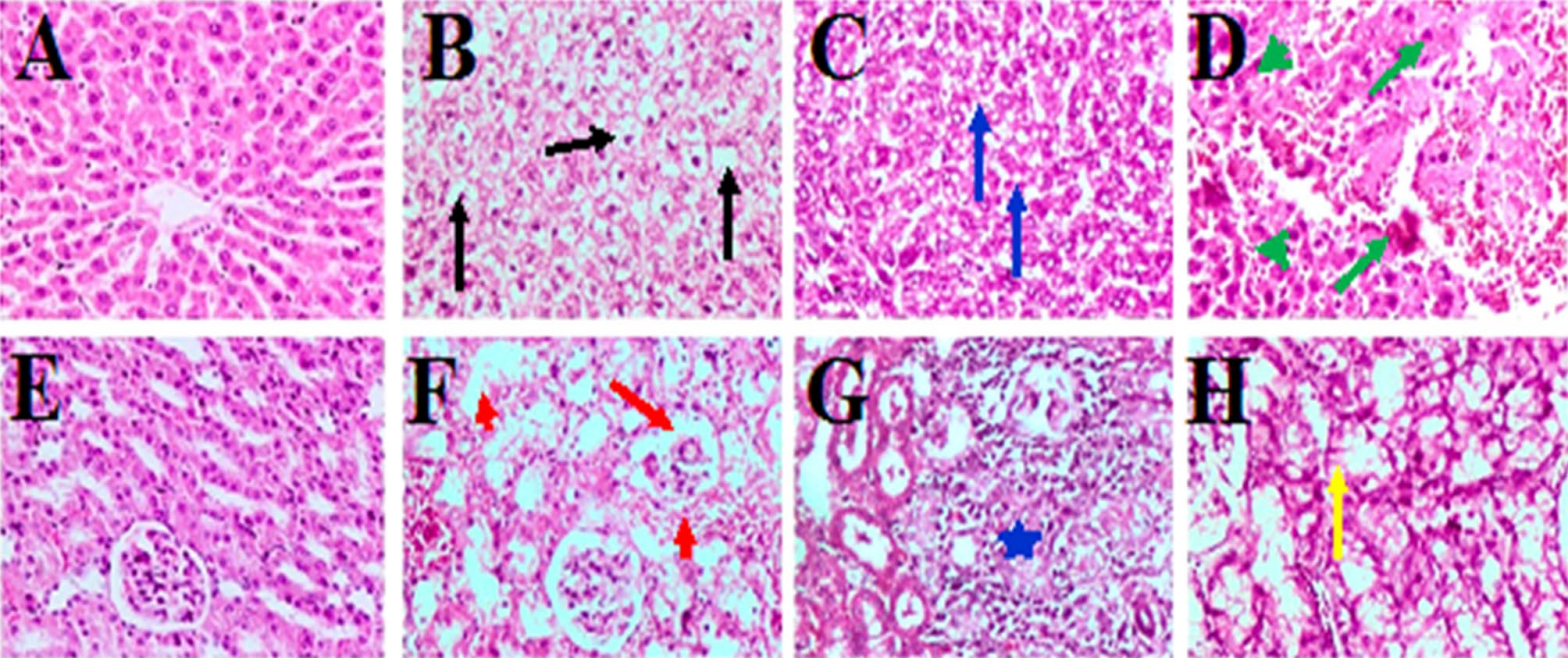
Photomicrographs of the liver (**A**–**D**) and kidney (**E**–**H**) from rats of groups C, nCB, CB10, and CB40, respectively, (H&E, × 200). Diffuse vacuolar degeneration of hepatocytes (black arrows), diffuse and mild to moderate fatty change (blue arrows), extensive necrosis of hepatic cells (green head arrow) associated with hemorrhage (green arrows), atrophy and shrinking of glomerular tuft (red arrow), necrosis of tubular epithelium (red short arrow), focal area of severe necrosis of renal tubules associated with infiltration of mononuclear cells (blue asterisk), severe vacuolar degeneration of epithelium lining of renal tubules (yellow arrow).

**Figure 4:**
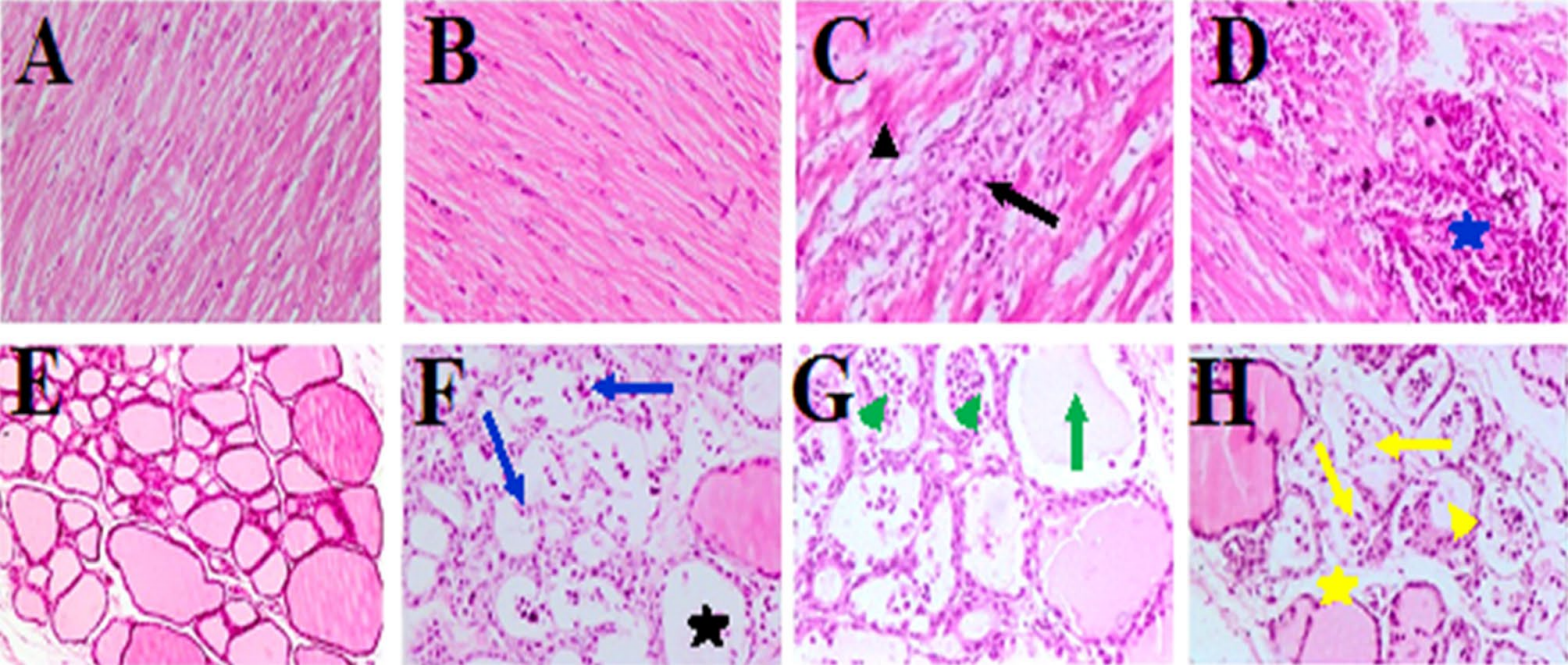
Photomicrographs of the heart (**A**–**D**), and thyroid glands (**E**–**H**) from rats of groups C, nCB, CB10, and CB40, respectively. All photos are (H&E, × 200) except Thyroid gland in photos (C) and (CB40) are (H&E, × 100) Aggregations of inflammatory cells (black arrow), hyaline degeneration (black head arrow), and severe interstitial hemorrhages (blue asterisk), exfoliation of necrotic follicular cells (blue arrow), empty or scanty amount of colloid (black asterisk), follicles are filled with desquamated follicular cells (green head arrows), with empty or scanty colloid (green arrows), severe necrosis of follicles (yellow arrow), with rupture of follicular wall and no colloid (yellow head arrow), and wide interfollicular space (yellow asterisk).

No microscopical changes were detected in the liver and kidneys (Fig. 3A,E), respectively, and in the control’s heart and thyroid glands (Fig. 4A,E), respectively. In the nCB group, the liver displayed the diffuse vacuolar degeneration of hepatocytes throughout the hepatic parenchyma and several areas with a mild fatty change in hepatic cells (Fig. 3B). In a few cases, the hepatic cords were disorganized and necrosed, dilating the hepatic sinusoids and bleeding. In the kidney, necrotic alterations in the epithelial lining of renal tubules are associated with atrophy and shrinking of some glomerular tufts of renal glomeruli (collapsed glomeruli) with dilatation of capsular space was seen (Fig. 3F). Furthermore, homogeneous eosinophilic material (renal casts) was observed within the lumen of some tubules. At the same time, a typical histological structure of the cardiac muscle was seen in the heart (Fig. 4F). Furthermore, the thyroid gland showed severe histological abnormalities in more than half the number of the animal group, indicating necrosis of the follicle’s epithelial lining, which resulted in exfoliation and desquamation of follicular cells within the lumen of follicles (Fig. 4F). Moreover, many follicles appeared involuted (atrophied and shrunken) and small in size with an empty or minimal amount of colloid. Meanwhile, other follicles showed vacuolation in their cytoplasm. Cystic follicular formation with flattening of epithelium lining and fusion of follicles as a product of basal lamina rupture was noticed. Hyperemia was present in the blood vessels.

In rats of group CB10, mild to severe fatty changes associated with hemorrhage among hepatic cells were seen in the liver (Fig. 3C). In some cases, the hepatic cords were necrotic and fragmented, with marked dilatation of hepatic sinusoids and the presence of erythrocytes. In the kidney, degenerative changes, mainly vacuolar type of the epithelium lining of renal tubules, were observed. In a few cases, there was a focal area of severe necrosis of renal tubules associated with infiltration of mononuclear inflammatory cells, primarily lymphocytes and macrophages, and renal casts within the lumen of some tubules (Fig. 3G). However, multiple foci of aggregations of mononuclear cell infiltration in the heart were seen. Occasionally, mild hyaline degeneration of myofibers was observed (Fig. 4C). Clusters of inflammatory cells were also found in the subpericardium. Thyroid glands showed irregularly shaped and sized follicles with no or very little colloid. Some follicles appeared to necrosis with desquamation of follicular epithelial cells in the lumen (Fig. 4G). Some follicles showed vacuolated cytoplasm. Others had multiple layers of follicular cells lining them. In rats of group CB40, the liver showed congestion in the blood capillaries between the follicles and moderate fatty changes throughout the hepatic parenchyma. In a few cases, there were also multiple areas of extensive hepatic cell necrosis associated with hemorrhage (Fig. 3D). The kidney showed severe vacuolar degeneration of the epithelium lining of renal tubules (Fig. 3H). Moreover, in a few cases, there was a focal area of necrosis of the epithelium lining of renal tubules associated with interstitial infiltration of inflammatory cells, especially around the renal blood vessels. In addition, massive hemorrhages among the renal tubules were observed. Occasionally, multiple foci of mononuclear cell aggregation were observed in the heart, besides mild hyaline degeneration of myofibers. Clusters of inflammatory cells were also discovered in the subpericardium. Furthermore, the heart sections of rats of the CB40 group exhibited severe interstitial hemorrhages in the myocardium (Fig. 4D). In addition, dilatation and congestion of blood vessels were observed. Moreover, there was a focal accumulation of inflammatory cells in a few cases. In the thyroid gland, irregularly shaped and sized follicles with absent or scanty amounts of colloid were seen (Fig. 4H). Some follicles showed necrosis with desquamation of follicular epithelial cells in the lumen and vacuolated cytoplasm, and some were lined by more than one layer of follicular cells. Congested blood capillaries in between follicles were also observed. Furthermore, it showed disorganization and damage of most follicles (necrosis) associated with the exfoliation of a follicular cell into the lumen and vast inter-follicular space. Some follicles revealed rupture of a follicular wall with coalescence of follicles. Meanwhile, other follicles were empty with no colloid in their lumina. Moreover, some follicles exhibited hyperplasia (stratification) of the epithelial lining.

**Figure 5:**
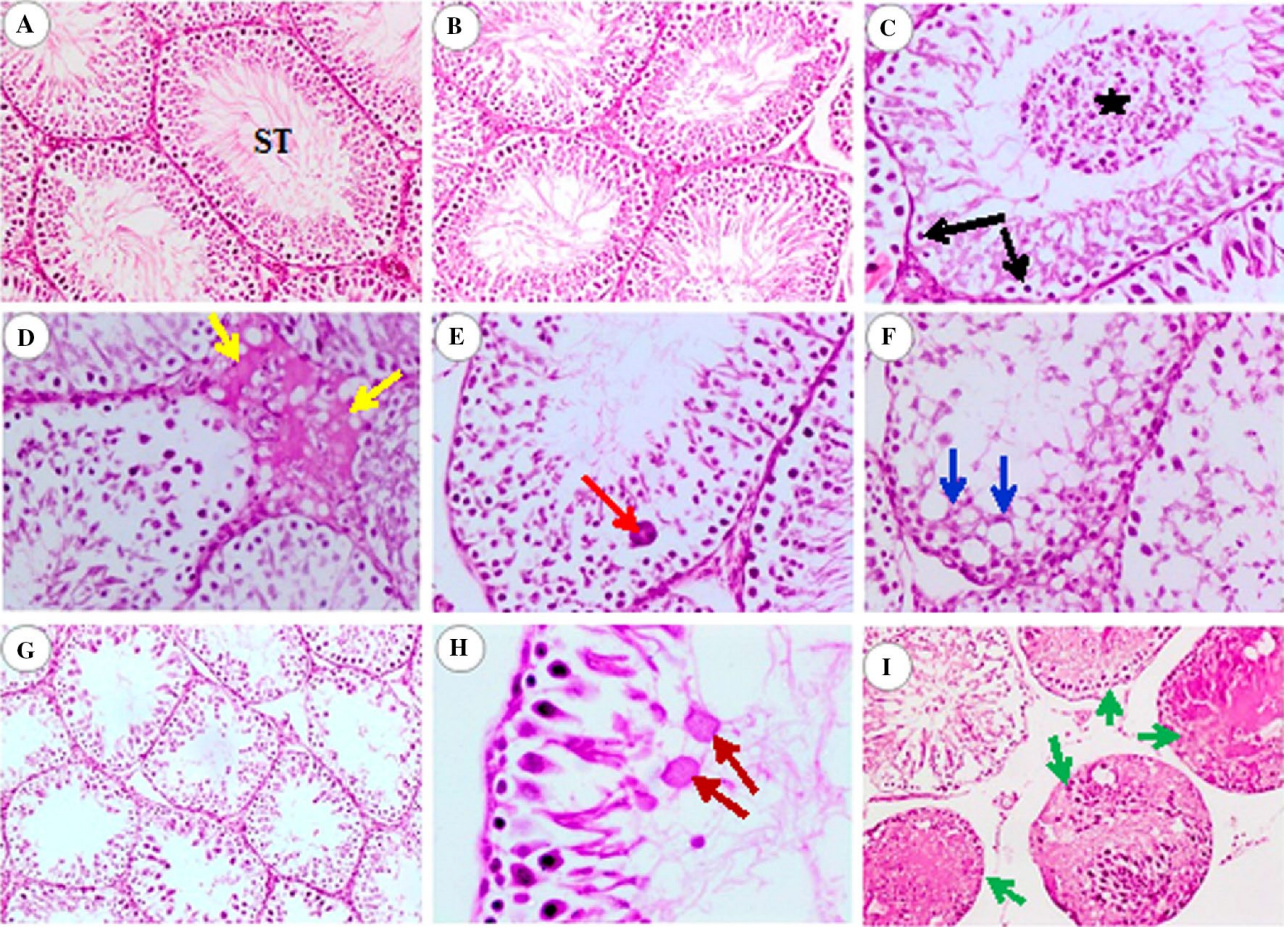
Photomicrographs of testes of rats; (**A**) control group, (**B**) nCB group, (**C**–**E**) CB10 group, and (**F**–**I**) CB40 group. Photos: (**A**,**B**,**G**,**I**) are (H&E, × 100), (**C**,**D**,**E**,**F**) are (H&E, × 200), and H is (H&E, × 400). Seminiferous tubules (S.T.), vacuolar degeneration, pyknosis of nuclei (black arrows), exfoliation of necrotic spermatogenic cells into the lumen (black asterisk), focal interstitial oedema (yellow arrows), multinucleated spermatid giant cell within the lumen of S.I. (red arrow), severe vacuolar degeneration, pyknosis of spermatogenic nuclei (blue arrows), eosinophilic structureless globules of coagulated spermatids (brown arrows), homogenous hyalinized eosinophilic material with no sperms within the lumen of ST (green arrows).

In both groups C and nCB, testes showed the typical histological structure of active functioning seminiferous tubules (ST) associated with normal spermatogenesis (Fig. 5A,B). Rat testes in the CB10 group showed vacuolar degeneration, pyknosis of spermatogenic nuclei, mild to moderate spermatogenic cell depletion, and exfoliated necrotic cells into the lumen (Fig. 5C). Furthermore, there was marked focal interstitial edema associated with blood vessel dilatation and congestion (Fig. 5D) and the presence of multinucleated giant cells within the lumen of ST (Fig. 5E). Testes of rats for group CB40 revealed severe vacuolar degeneration and pyknosis of spermatogenic nuclei (Fig. 5F). Moreover, most of the STs showed moderate to severe depletion and reduced the number of spermatogonial layers lining the ST (Fig. 5G). Many multinucleated giant cells desquamated spermatogenic cells, and cellular debris within the lumen of some ST was observed. Coagulation of spermatids in eosinophilic structureless globules was seen (Fig. 5H). At the same time, several tubules contained homogenous hyalinized eosinophilic material with no sperms within the lumen (Fig. 5I). Leydig cells appeared vacuolated and proportionally increased in number. Prominent focal interstitial edema among the tubules and congestion of blood vessels were also seen.

## Discussion

In animal studies, the most common route of NP administration is oral, inhalation, and injection (either subcutaneous or intraperitoneal). Due to their small size, nanoparticles can enter the circulatory and lymphatic systems and then into the tissues and organs. Many studies proved that the main focus organs after oral administration for engineered NPs are the liver, kidneys, and spleen^44^.

Moreover, irreversible damage to cells occurred through oxidative stress and/or organelle injury due to several factors, including the size, shape, composition, and surface chemistry of the NPs^45^. The first experiment revealed a nonsignificant difference in FBW and BWC of G2 and G3 compared to the G1 (Table 1). In addition, we established that all groups lost weight when comparing FBW to IBW. Furthermore, there are nonsignificant changes in the relative weights of the liver, kidneys, spleen, testes, and heart between the G1 and G3 (Table 1).

This finding was consistent with Asadi et al.^46^ who discovered that rats administered with MoO_3_-NPs had a lower body weight when compared to the body weight of the mice in the control group. Furthermore, exposure to MoO_3_-NPs resulted in a 3% decrease in body weight compared to the weight of the animals at the start of the experiment. Moreover, the MoO_3_-NPs effectively inhibited weight gain during short-term treatment^47^. Bodyweight is a sensitive indicator that is affected by toxic chemicals. Moreover, gastrointestinal disorders, lack of appetite, and delayed absorption of foods are significant factors that lead to weight loss^48^. The decrease in the relative weight of organs could be attributed to either the loss of body weight, which eventually affected most organs such as the liver, spleen, and testes, or to the significant impact of the NPs on these organs.

In the first experiment, we discovered a significant increase in ALT, albumin, and total protein levels, besides a nonsignificant increase in UA in G3 compared to the respective levels in G1. Compared to control, the plasma urea levels significantly increased in G2 (Table 2). Meanwhile, when compared to the G1, G3 showed a significant decrease in ALP, LDH, creatinine, CK-MB, TSH, FT3, and testosterone. Furthermore, when compared to G1, most biochemical analyses revealed a nonsignificant change in G2.

In contrast, Asadi et al.^46^ suggest that MoO_3_-NPs are hazardous to AST, testosterone, and LDH levels in the serum. According to histological inspection, the number of chronic inflammatory cells in the portal triad was gradually higher in the liver tissue sections of rats exposed to Mo-NPs, and there was an increase in apoptotic cells. Hepatic cells showed no evidence of necrosis or vacuolation.

This study established that the rise in urea levels in G2 could be attributed to the breakdown of the kidney’s tissue structure after Mo nanoparticles were administered. Tang et al.^49^ also discovered that ZnO nanoparticles could considerably increase urea and creatinine levels. In this regard, Xiao et al.^50^ found damage in the renal cells and apoptosis in the kidney in broilers fed Mo nanoparticles at doses ranging from 13 to 1500 mg/kg body weight.

In contrast to our results, Sizovaet al.^51^ showed no change in the urea and creatinine levels at lower and higher doses of MoO_3_-NPs and no change in the activity of LDH at the low dose of MoO_3_-NPs, while at a high dose, there was a rise in the activity of AST and LDH, indicating that MoO_3_-NPs are moderately toxic when compared to other transition metals. Workers at a molybdenite roasting plant exposed to Mo had high UA levels in their blood and clinical signs that resembled osteogenesis defects and gout-like illnesses^52^.

In contrast to our findings, Asadi et al.^47^ found that when MoO_3_-NPs groups were compared to control groups, there was a significant rise in serum TSH levels but no change in the T3 levels. Consistent with our findings, Asadi et al.^47^ found that the oral exposure of MoO_3_- NPs (10 and 15 mg/kg BW) for 1 week in rats led to damaged testes and reduced Leydig cell number. Furthermore, the diameter of the seminiferous tubule was reduced compared to control rats. In addition, testosterone levels appeared to be significantly reduced in MoO_3_-NPs groups than in the control group.

Nanoparticles can negatively affect and decrease testosterone secretion in the cells by exerting disruptive effects on mitochondria^53^. Another possible mechanism is that NPs release reactive oxygen molecules, which can cause cell damage and death, leading to a decrease in testosterone-producing cells^54^. Moreover, a reduction in steroidogenic acute regulatory gene expression due to exposure to NPs could cause a decrease in testosterone production. The product of this gene is a carrier protein transporting cholesterol to the inner cell membrane of mitochondria for steroidal hormone production^55^. In addition, Pandey et al.^56^ discovered that instantaneous Mo ingestion could alter sperm morphology.

Compared to the control group, the number of WBCs in G2 and G3 was significantly lower, while the number of WBCs in G3 was significantly higher (Table 3). Asadi et al.^46^ discovered that the number of WBCs increased as the dosage of Mo-NP increased, which is consistent with our findings. However, NPs enter the lymphatic system at high concentrations and cause inflammation. As a result, the induced inflammation could be responsible for increasing WBC counts^57^. The reduction in the number of WBCs could be due to the increased concentration of nanoparticles, which causes more contact surfaces and a more significant effect on the membrane of the cells, penetrates the white blood cell mitochondria, and changes the activity of their enzymes^58^. Nanoparticles also reduce cellular activity, raise oxidative stress, and minimize the antioxidant activity of the cell, resulting in a decrease in the number of WBCs^59^. In addition, as compared to the control group, G3 had a considerable increase in RBC, hemoglobin, and hematocrit. Compared to the control group, oral administration of MoO_3_-NPs (10 or 40 ppm) did not affect RBC indices in the first trial (Table 3). This finding is in line with Asadi et al.^46^, who found no significant influence on the RBC indices. In contrast, Fazelipour et al.^60^ found that experimental groups treated with MoO_3_-NPs had higher MCV and MCH values and lower MCHC.

Our findings from the second experiment revealed that the FBW of animals in all groups increased at the end of the treatment when compared to their IBW (Table 4). All CB groups, had significantly higher FBW, BWC, and TFI than the control group. Furthermore, compared to the control group, we found no change in the relative weight of the liver, heart, and testes in all CB groups. While, the relative weight of the spleen was nonsignificantly decreased in the nCB and CB40 but significantly decreased in the CB10 group. Furthermore, the kidney’s relative weight was significantly lower in all CB groups than in the control group (Table 4).

When comparing the two treated groups (CB10 and CB40) to the control group, we discovered a substantial rise in AST in the nCB group and a nonsignificant increase in AST in the two treated groups. In addition, all CB groups showed a significant reduction in ALT than the control group (Table 5). Asadi et al.^46^ found that the serum levels of AST reduced considerably at doses of 5 and 10 mg/kg BW of Mo-NPs. However, ALT nonsignificantly increased in the Mo-NPs group that received 10 mg/kg BW for 28 days than in the control group.

Furthermore, all CB groups showed a significant increase in ALP levels than the control group. Total protein was considerably lower in the CB10 and CB40 groups than in the control group, while LDH was much lower in the CB10 and CB40 groups. Furthermore, the nCB group had a significant growth in urea and creatinine, besides a nonsignificant increase in UA compared to the control. This increase was improved in the CB10 and CB40 groups than the nCB group (Table 5).

UA is the byproduct of purine metabolism. Most purines are derived from endogenous synthesis, but a minor portion is derived from exogenous sources such as purine-containing foods, alcohol, and fructose drinks. Non-modifiable factors influence serum UA concentrations, while modifiable variables like body weight and diet and its purine content play a significant role^61^. This could explain the rise in UA levels in all CB groups. Molybenum serves as a cofactor for the enzyme xanthine oxidase, which catalyzes the oxidation of purines to UA^62^.

Also, the blood glucose levels were considerably higher in the CB10 and CB40 groups than in the control group (C), but lower when compared to the nCB group. This is because Mo enhances glucose homeostasis in hepatocytes, according to Reul et al.^63^, by increasing the insulin receptor tyrosine kinase activity. TSH and FT3 were significantly lower in the CB10 and CB40 groups than in the control group.

The results exhibited a significant drop in the serum level of testosterone in CB10 and CB40 groups than in the control group. This decrease was dose-dependent, agreeing with Pandey and Jain^64^ findings. Meeker et al.^65^ showed a decline in testosterone levels after Mo exposure in a case study. The decline in testosterone levels in the rats nourished on CB fertilized by MoO_3_-NPs could be attributed to a reduction in the pituitary LH secretion or underutilization of cholesterol in testosterone synthesis^64^. Increased oxidative stress could potentially affect the Leydig cells' ability to operate normally. In molybdenum-treated sheep^66^, rats^64^, and rabbits^67^, histopathological checking of the testes exposed an elevated attribution of deteriorated ST, with the marked infirmity of the lining epithelium and reduced spermatogenesis.

NPs are absorbed into the bloodstream, dispersed in the body, and eliminated in the urine. Otherwise, the liver and kidney could be potential target organs for MoO_3_-NPs accumulation and toxicity. Hematological parameters are also vital in appraising the toxicity of MoO_3_-NPs^68^. Nanoparticles should be avoided because they can cause immunotoxicity when interacting with the immune system^69^. As a result of either direct toxicity or immune-mediated injury produced by drugs, the number of neutrophils has been demonstrated to decrease^70^.

Furthermore, we discovered a significant decrease in platelet count in the CB10 and CB40 groups than in the control, which is consistent with Akhondipour et al.^71^. We also detected a significant difference in RBC, hemoglobin, and hematocrit in both treated groups than in the control group in the second experiment. According to Rezaei-Zarchi^72^, nanoparticles are accountable for altering platelets’ integrin and phosphoprotein levels. The researchers described the mechanism of nanoparticle influence on the platelet, a process in which nanoparticles enter platelets and occupy spaces and ecovolar granules, preventing the growth of hyaloplasmic and reducing platelet aggregation^73^. Furthermore, we discovered a significant drop in granulocytes when comparing the CB10 and CB40 groups to the control group. Other hematological parameters did not significantly alter (Table 4).

According to several animal studies, high quantities of Mo have interfered with copper metabolism in animals, causing a change in their typical hematological profile, according to several animal studies^74^. Sobańska et al.^75^ established that the NPs might induce a protective reaction in the cells, stimulating the antioxidative response and reducing its toxic effect. Hence, the low cytotoxicity would be a matter of complicated cellular balance^76^. Histological examination of the liver, kidney, heart, and testicular tissue confirmed our biochemical findings.

## Conclusion

According to our findings, exposure to MoO_3_-NPs had a negative impact on the liver, kidney, heart, and testes. High doses of MoO_3_-NPs influenced the liver, kidney, and heart functions, as well as blood glucose levels, hormone levels, and hematological parameters, either by direct oral exposure to MoO_3_-NPs or by eating CB fertilized by MoO_3_-NPs. A histopathological examination of the liver, kidney, heart, thyroid gland, and testes confirmed our findings.
